# Analyzing the miRNA-Gene Networks to Mine the Important miRNAs under Skin of Human and Mouse

**DOI:** 10.1155/2016/5469371

**Published:** 2016-09-05

**Authors:** Jianghong Wu, Husile Gong, Yongsheng Bai, Wenguang Zhang

**Affiliations:** ^1^College of Animal Science, Inner Mongolia Agricultural University, Hohhot 010018, China; ^2^Inner Mongolia Academy of Agricultural & Animal Husbandry Sciences, Hohhot 010031, China; ^3^Inner Mongolia Prataculture Research Center, Chinese Academy of Science, Hohhot 010031, China; ^4^State Key Laboratory of Genetic Resources and Evolution, Kunming Institute of Zoology, Chinese Academy of Sciences, Kunming 650223, China; ^5^Department of Biology, Indiana State University, Terre Haute, IN 47809, USA; ^6^The Center for Genomic Advocacy, Indiana State University, Terre Haute, IN 47809, USA

## Abstract

Genetic networks provide new mechanistic insights into the diversity of species morphology. In this study, we have integrated the MGI, GEO, and miRNA database to analyze the genetic regulatory networks under morphology difference of integument of humans and mice. We found that the gene expression network in the skin is highly divergent between human and mouse. The GO term of secretion was highly enriched, and this category was specific in human compared to mouse. These secretion genes might be involved in eccrine system evolution in human. In addition, total 62,637 miRNA binding target sites were predicted in human integument genes (IGs), while 26,280 miRNA binding target sites were predicted in mouse IGs. The interactions between miRNAs and IGs in human are more complex than those in mouse. Furthermore,* hsa-miR-548*,* mmu-miR-466*, and* mmu-miR-467* have an enormous number of targets on IGs, which both have the role of inhibition of host immunity response. The pattern of distribution on the chromosome of these three miRNAs families is very different. The interaction of miRNA/IGs has added the new dimension in traditional gene regulation networks of skin. Our results are generating new insights into the gene networks basis of skin difference between human and mouse.

## 1. Introduction

The integument includes the skin, coat/hair, and nails [1]. The mammalian coat hair is one of the defining characteristics of mammals [2]. Enormous morphological variations are found among mammalian integument, especially in coat hair. Hair is present in differing degrees in all mammals [3]. In most mammals, the hair is abundant enough to cover the body and form a thick coat, while dolphins, naked mole-rats, and humans are among the most hairless of all mammals [4]. However, humans with the hypertrichosis syndrome have hair covering their faces, their eyelids, and their bodies [5, 6]. Therefore, we provide a hypothesis that key genes related to hair coat formation exist in every mammalian species. Mammals have diverse coat morphology, due to changes in the gene regulatory pathway/network. Common genetic network variants have been shown to affect many complex traits, including integument morphology. Therefore, to understand the differences of molecular mechanism for integument phenotype between mouse and human, it is necessary to employ the scope of system biology. The comparison of gene coexpression networks and regulation networks between two species is often a useful method to identify the differences of critical biologic process associated with morphology diversity.

Mammalian Phenotype (MP) ontology [7] (http://www.informatics.jax.org/) is made up of 17,330 terms (as of 02/08/2016), and most of these terms were characterized from abnormal mouse phenotypes. The ontology database deposited 1627 mouse/human orthologous genes with phenotype annotation related with integument (MP:0010771). High-throughput experiments have produced many microarrays and next generation sequencing data that are collected in Gene Expression Omnibus (GEO) database [8]. Genetic networks have been widely used in biological research, bridging the gap between single genes and biological systems by investigating the relationships between different genes.

miRNAs are small noncoding RNA molecules which play an essential role during skin development [9]. Variations in the interaction between miRNA and target gene are likely to influence the phenotypic differences between species. Here, we use Weighted Gene Co-Expression Network Analysis (WGCNA) to reveal shared and unique characters of the gene networks in human and mouse skin. We identify networks of coexpressed genes, which might be associated with biological functionally relevant coat morphology, and explore differences in these biological processes between two species. We also found that candidate miRNA may play a role in the regulation of gene expression networks in skin. These results provide a system-level insight into evolutionary changes of the integument.

## 2. Materials and Methods

In the present study, we emphasize the comparison between the gene network for integument of human and that of mouse and identification of candidate miRNAs relevant to the integument genetic pathway and constructed an interaction network between miRNAs and targeted genes. Figure S1 in Supplementary Material available online at http://dx.doi.org/10.1155/2016/5469371 shows pipeline for the study.

### 2.1. Selection of Genes and miRNAs for Human and Mouse

Mouse/Human Orthology with Phenotype Annotations were downloaded from the MGI database [10]. 1627 of these genes are related to the integument phenotype (MP:0010771). To draw the chromosome location for integument genes (IGs), the R package org.Mm.eg.db [11] and org.Hs.eg.db [12] were used. We downloaded the gene expression data of skin for mouse and human from the Gene Expression Omnibus (GEO) [13] and mouse and human miRNAs from miRBASE (http://www.mirbase.org/cgi-bin/browse.pl/) [14]. Mouse and human 3′UTR sequences of 1627 integument genes were obtained from Ensemble (http://www.ensembl.org/) by biomaRt in R language [15].

### 2.2. Construction of Gene Networks

To compare the gene networks of skin for human and mouse, we selected many relevant data from the GEO database [8], and then we filtered out all but a core collection of datasets that were similar enough for useful bioinformatic comparison. First, we downloaded all datasets that were run on same Affymetrix platform, one platform in human (GPL570) and one in mouse (GPL1261) (Table S1). Second, we selected only relevant experiments about skin samples on the platform. Third, we extracted IGs expression data from the chip matrix. Finally, a total of 1487 genes were analyzed in this study. Then, Weighted Gene Co-Expression Network Analysis (WGCNA) [16, 17] was used for these expression datasets to create consensus networks between human and mouse, and the networks were visualized in Cytoscape 3.3.0 [18].

### 2.3. Functional Enrichments for IGs Network

To classify the terms and group for IGs in gene ontology (GO) and KEGG pathway, we used the Cytoscape plug-in ClueGO to implement enrichment analysis. We set kappa score threshold to 0.5 and the *p* value to 0.05; we used two-sided test, Bonferroni step down and GO term fusion. The other parameters of the software were set to default values [19].

### 2.4. Prediction of miRNA Targets for Integument Genes

For prediction of miRNA targets for hub genes, miRanda [20] software version aug2010 available at http://cbio.mskcc.org/microrna_data/miRanda-aug2010.tar.gz was employed to predict miRNAs regulated IGs. miRanda was running as the following command options: sc ≥ 180, en = 1 (no energy filtering), go = −9.0, ge = −4.0, and scale = 4.0. We set the option* -strict* to prevent gaps and noncanonical base pairing in target sites. Human/mouse miRNA sequences were used as query sequences, and the IGs sequences were used as [20]. We also predicted the targets with two miRNA prediction databases, miRTarBase [21] and miRDB [22], and selected the intersections targets to construct the miRNA-mRNA regulatory network.

## 3. Results

### 3.1. Chromosome Location for Integument Genes of Human and Mouse

We observed a uniform distribution of the 1627 integument genes across all chromosomes in human and mouse (Supplementary Figure S2). As can be seen in Figure S1, in general, 1627 integument genes are dispersed along the chromosomes of human and mouse. These results provide insights into the biological underpinnings of integument phenotype and the pleiotropic connections between traits. It is very difficult to detect which chromosome is essential to integument phenotype because these integument genes will be uniformly distributed on all chromosomes.

### 3.2. Gene Expression Network

**Figure 1:**
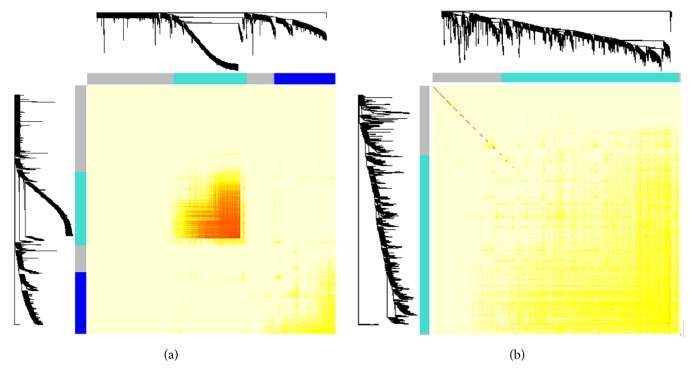
The TMO plot shows the modules of gene expression in human (a) and mouse (b) by WGCNA.

**Figure 2:**
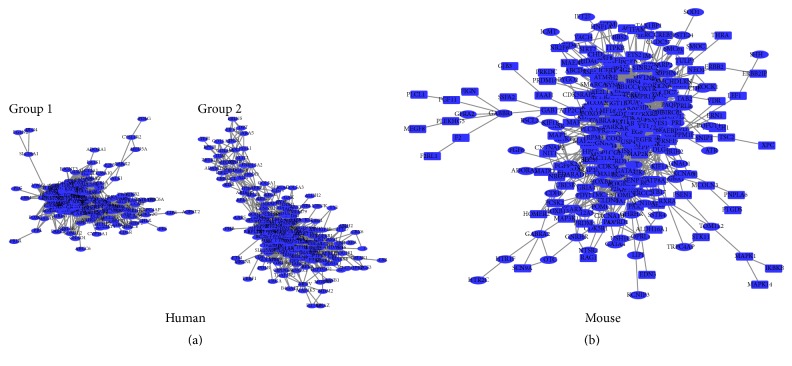
The consensus networks for skin of human and mouse: (a) for human and (b) for mouse.

By using WGCNA, we generated two consensus networks of the human and mouse (Figure 1) which show a network heat map plot of a gene network together with the corresponding hierarchical clustering dendrograms and the resulting modules. Then, the gene expression networks were visualized in Cytoscape. There are two groups in human skin consensus networks constructed from 560 genes (nodes) and 18391 interactions (edges), while only one group in mouse skin gene networks was constructed from 368 genes and 1757 interactions (Figure 2). To evaluate the complexity of gene networks, we calculated the interaction degrees for gene expression network. The average interaction degrees are 32.8 (18391/560) and 4.8 (1757/368) for human and mouse, respectively.

**Figure 3:**
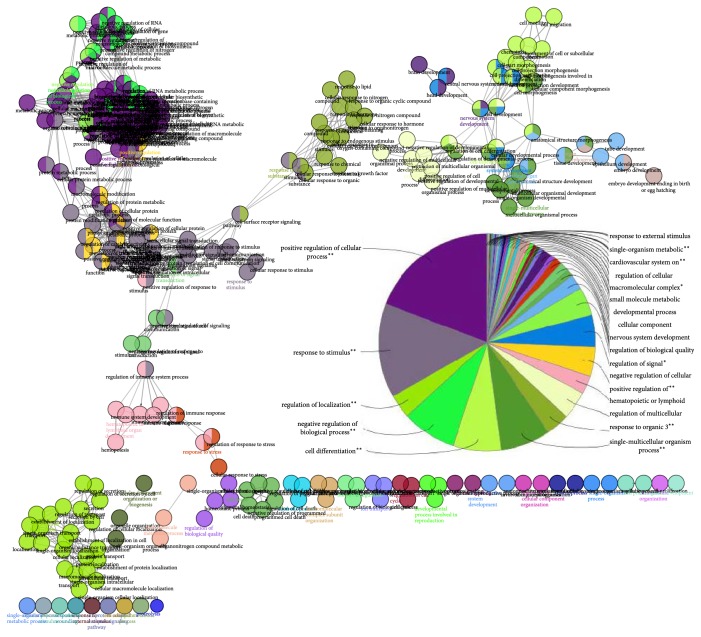
Overrepresented GO categories in the integument gene list of human and mouse. The figure was drawn by Cytoscape 3.3.0 and shows enriched GO categories for common genes in three clusters. The pie chart shows the leading group term in each of the GO categories. The kappa score threshold was set to ≥0.5. ^*∗∗*^If the term/group is oversignificant (*p* value < 0.001). ^*∗*^If the term/group is significant (0.001 < *p* value < 0.05).

We use the ClueGO to compare three clusters of genes from Group 1 and Group 2 of human and the mouse consensus networks (Figure 2). 44 significantly overrepresented categories were identified (Figure 3). Most of the positive regulations of cellular process were clustered into two independent modules. More than half of the common genes among three clusters were involved in regulation of cellular and metabolic processes, such as negative regulation of nucleobase-containing compound metabolic process and regulation of macromolecule biosynthetic process. Some of the most significant categories were response to stimulus, regulation of localization, negative regulation of biological process, cell differentiation, single-multicellular organism process, and response to organic substance. Analysis of the modules showed that the majority of the response to stimulus genes and response to organic substance were common in three clusters.

**Figure 4:**
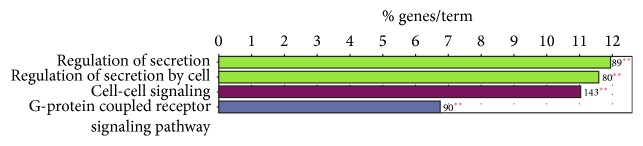
Specific GO term enrichment as estimated for the human versus mouse. ^*∗∗*^If the term/group is oversignificant (*p* value < 0.001). ^*∗*^If the term/group is significant (0.001 < *p* value < 0.05).

Furthermore, we found that four GO terms were highly enriched, and these terms were specific in human compared to mouse. These four GO terms could be divided into three categories (Figure 4). The regulation of secretion category contains two GO terms: regulation of secretion and regulation of secretion by cell.

### 3.3. miRNA Target Sites in IGs

**Figure 5:**
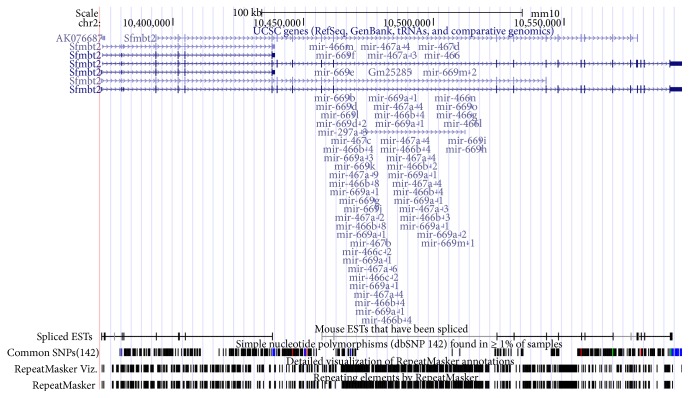
The chromosome location of mmu-miR-466 and mmu-miR-467 family.

**Figure 6:**
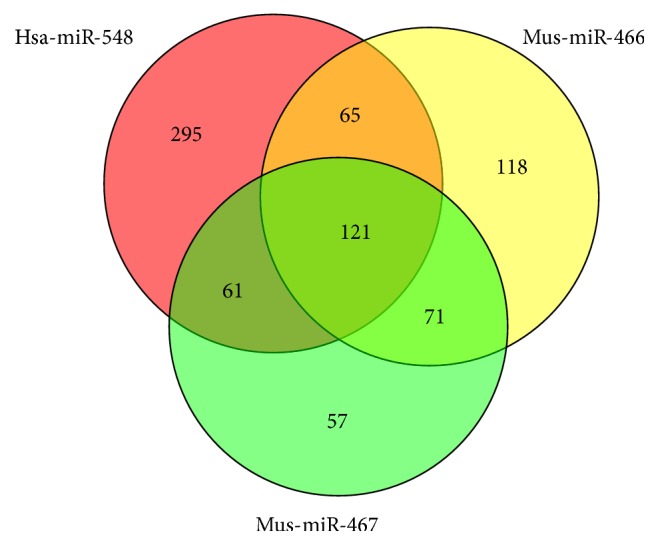
The Venn diagram of the target gene list of three miRNAs.

miRNAs are implicated in integument development. To examine how the miRNAs interacted with the gene networks under integument, we used miRanda to predict the miRNA targets for IGs. Total 62,637 miRNA binding target sites were predicted for 1627 human IGs selected and the dataset of 2588 human miRNAs used for the analysis. Total 26,280 miRNA binding target sites were predicted for 1627 mouse IGs selected and the dataset of 1915 mouse miRNAs used for the analysis. A single miRNA can regulate hundreds of transcripts and a single transcript can have binding sites for multiple miRNAs of the same or different sequence. To determine the portion of the main miRNA in the total miRNA target file, we selected the top 10 miRNA families based on the target number of IGs (Table 1). The* hsa-miR-548* family has predicted 4643 target sites distributed on 541 IGs of the human. The* mmu-miR-466* family and* mmu-miR-467* family have predicted 1,704 target sites and 956 target sites, respectively, distributed on 375 and 310 IGs of the mouse. The* hsa-miR-548* family is widely distributed in the whole human genome. However, both of these two miRNA families are located in an intron of* Sfmbt2* gene on chromosome 2 of the mouse (Figure 5). These three miRNA families have common target genes (Figure 6) and might present some similarity function to skin morphogenesis.

### 3.4. Construction of a miRNA-mRNA Regulatory Network

**Figure 7:**
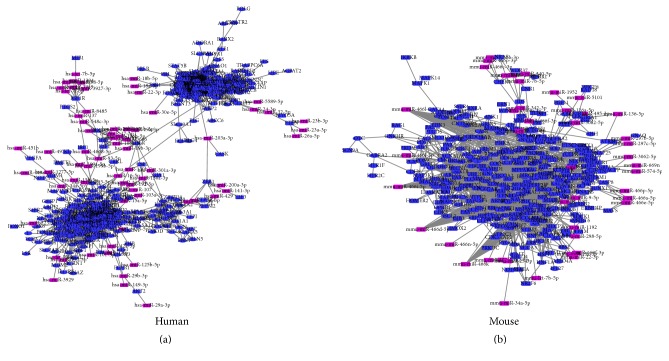
The interaction networks between IGs and miRNA family. (a) For human and (b) for mouse. The purple nodes represent miRNAs and the blue nodes represent target gene.

Two miRNA prediction databases miRTarBase and miRDB were used to verify the results of miRanda. The intersections targets of three algorithms were selected to construct the miRNA-mRNA regulatory network (Table S2). The resulting miRNA/target mRNA pairs and gene networks for human and mouse were visualized in Cytoscape, where edges from miRNA to genes represent a potential regulatory relationship and edges from gene to gene represent an expression correlation. The network consisted of two almost separate groups, which could be connected when* miRNAs* is added to the expression gene network of human (Figure 7). These miRNA-target interactions* were* added to the network of the mouse, which have not significantly affected the consensus networks in mouse skin (Figure 7).

## 4. Discussion

Recently, a lot of biology databases have been published. How to integrate these resources in system biology is a big challenge for analysis of certain biological problems [23]. In this study, we have integrated the MGI, GEO, and miRNA databases to analyze the genetic regulatory networks under morphology difference of integument of human and mouse. 1627 mouse/human orthologous genes related with integument phenotype were deposited in MGI database. These genes were widely scattered across the mouse genome and the human genome, which suggest that the integument is a quantity phenotype with polygenic determinism. We also have constructed the expression correlation networks of IGs with the gene expression matrix from the GEO database. With the process of evolution, the organisms have increased in complexity. However, the organism's complexity is not simply associated with the number of its genes [24]. Now, the interaction degrees as a straightforward detector were used to evaluate the complexity of gene networks [25]. Our results revealed that the networks of human skin are much more complex than those of mouse which might be explained by the fact that the integument structure at the anatomical level is much more complex in human, compared with mouse [26]. Many researches reported that one gene could evolve new function to adapt to the change of environment during species divergence, from lower to higher species [27, 28]. Erwin and Davidson reported that the reorganizations of gene networks could change the animal morphology and the basic of the evolution is regulatory changes within a gene regulation network [29]. These results also confirm our hypothesis that those key genes related with the hair coat exist in every mammalian genome and the diverse morphology in mammal just because of the difference in gene regulation networks.

Analysis of the GO terms category showed that the majority of the positive regulations of cellular process and response to stimulus genes were common in three clusters, which may be due to the perception function of skin to in vivo and in vitro environments [30, 31]. In other words, these common categories in human and mouse skin were involved in protection [32, 33], sensation [34], temperature regulation [34], immunity [35], exocrine, and endocrine. By comparing the GO terms for those genes in expression network, the term of regulation of secretion category was enriched in human skin. Human has eccrine glands and thermal apocrine glands. Apocrine glands are always associated with hair follicles, and eccrine sweat glands cover almost the entire body surface of human [36], while eccrine sweat glands in the mouse are found only on the footpads [37]. These results might provide an important insight into the evolution of thermal eccrine system in human. The secretion category of the genes might be involved in the eccrine system in human.

miRNA plays an essential role in the regulation of skin development and morphogenesis [38]. miRNA family is a group of miRNAs that share common seed sequences, which has a similar regulation function [39]. We found that the* hsa-miR-548* family has the highest amount of target sites among the identified miRNAs in human and the* mmu-miR-466* and* mmu-miR-467* families are top two in the miRNAs list predicted in the mouse. Generally, increasing the number of target sites within a gene improves the efficiency of miRNA regulation [40]. More target sites provide more opportunity for recognition by miRNA and increase the kinetics and overall level of transcript regulation [41].* miR-548 *is a primate-specific miRNA family, which has 69 members distributed in almost all human chromosomes [42]. The* hsa-miR-548* family is involved in multiple biological processes, such as signaling pathways, immunity, and osteogenic differentiation, and some cancers [43–47].* hsa-miR-548* takes part in* IFN *signaling which responds to the virus and bacterial infections on the cell [46].* hsa-miR-548* also can turn down the host antiviral response by degradation of* IFN-λ1* [43]. UVB irradiations can downregulate* hsa-miR-548* of human epidermal melanocytes cell [48]. These results suggested that* hsa-miR-548* might contribute to dynamic regulatory network of skin transcriptome of human. Compared to human,* mmu-miR-466 and 467* families only located in intron 10 of Sfmbt2 genes. This area of intron 10 has a larger cluster of miRNAs, which is specifically present in mouse and rat [49]. Based on miRBase database (http://www.mirbase.org/) definition, clustered miRNAs are a group of miRNAs located within 10 Kb of distance on the same chromosome. In this study,* mmu-miR-466*,* mmu-miR-467*, and* mmu-miR-669* clusters have one core promoter region and transcriptional start site shared with the Sfmbt2 gene. The expressions of* mmu-miR-466 and mmu-miR-467* markedly waved during the hair follicle cycling in mouse [50] and were downregulated in melanoma of mouse by curcumin diet [51]. And histone deacetylation and metabolic oxidative stress can induce the activity of* mmu-miR-466* [52]. Skin serves as a barrier between body and pathogens (disease causing organisms), which is part of the innate immune system [53]. Both of* hsa-miRNA-548* and* mmu-miR-466* and* mmu-miR-467* can inhibit the host immunity response [54]. It is necessary to carry out the research on how these miRNAs contribute to integument morphogenesis in the future. When we added the relationship of miRNAs and their target genes by three prediction algorithms to the expression correlation networks, those two clusters in gene regulatory networks of human have been integrated, which add a new dimension to genetic networks under human integument. The changes of the hub gene in gene regulatory networks result in the evolutionary alternation and the morphological difference [29].

## 5. Conclusions

Genetic networks variants have been shown to affect many complex traits, including integument morphology. In this study, we try to compare the regulatory networks of miRNAs and IGs in the skin of human and mouse. We downloaded mRNA expression data in human and mouse skin from the GEO database to create within-species consensus networks by WGCNA. There is a big difference in consensus networks between human and mouse; human consensus networks are more complex compared to mouse. Two principal regulatory networks were found in human: one module contains 286 IGs specifically involved in secretion, whereas the other module contains 250 IGs which are enriched for cellular response to stress and catabolic process. The secretion category, which is specific category for human compared to mouse, of the genes might be involved in eccrine system in human. Then, total 62,637 miRNA binding target sites were predicted in human IGs, while 26,280 miRNA binding target sites were predicted in mouse IGs. The interactions between miRNAs and IGs of human are also more complex compared to mouse. To further detect the role of these miRNAs, miRNA/IGs specific regulatory networks were added in IGs expression correlated networks, which will advance the dimension to skin regulation networks.* hsa-miR-548*,* mmu-miR-466*, and* mmu-miR-467* have an enormous number of targets on IGs, which both have the role of inhibition of host immunity response. The regulations of transcription factors to downstream genes and miRNA to transcription factors affect the spatial and temporal expression of genes during skin morphogenesis. Our results provide a new avenue to understand the genetic networks basis of skin difference between human and mouse.

## Supplementary Material

Figure S1: A summarizes this entire methodology. 1627 of these genes are related with the integument phenotype (MP:0010771). The expression data of 1487 IGs of mouse and human were downloaded from the Gene Expression Omnibus (GEO). MicroRNAs (miRNAs) that potential regulate IGs were predicted by miRanda, miRTarBase and miRDB. The regulation network of miRNAs-IGs and IGs-IGs was integrated, and the networks were visualized in Cytoscape. Figure S2: Chromosome location for integument genes, left for human and right for mouse. The distribution of IGs on chromosomes for human and mouse was investigated by two R packages (org.Mm.eg.db and org.Hs.eg.db). 1627 IGs are dispersed along the chromosomes of human and mouse. Table S1: The informations of microarray data used in this paper. Table S2: The common pairs of miRNA-target gene predicted by three databases (miRanda, miRTarBase and miRDB).

## Figures and Tables

**Table 1:** Top 10 miRNA families for IGs of human and mouse.

	Human miRNA family	Target number	Mouse miRNA family	Target number
1	hsa-miR-548	4643	mmu-miR-466	1704
2	hsa-miR-1273	547	mmu-miR-467(669)	956
3	hsa-miR-30	412	mmu-let-7	235
4	hsa-miR-520	332	mmu-miR-297	222
5	hsa-miR-302	281	mmu-miR-6951	176
6	hsa-miR-3689	277	mmu-miR-3473	173
7	hsa-miR-4763	273	mmu-miR-30	166
8	hsa-miR-619	266	mmu-miR-7116	154
9	hsa-miR-4775	262	mmu-miR-181	140
10	hsa-miR-513	241	mmu-miR-7661	133

## References

[1] Chernova O. F. (2009). Skin derivatives in vertebrate ontogeny and phylogeny. *Biology Bulletin*.

[2] Maderson P. F. A. (2003). Mammalian skin evolution: a reevaluation. *Experimental Dermatology*.

[3] Murphy W. J., Eizirik E., Johnson W. E., Zhang Y. P., Ryder O. A., O'Brien S. J. (2001). Molecular phylogenetics and the origins of placental mammals. *Nature*.

[4] Pagel M., Bodmer W. (2003). A naked ape would have fewer parasites. *Proceedings of the Royal Society of London B: Biological Sciences*.

[5] Zhu H. W., Shang D., Sun M. (2011). X-linked congenital hypertrichosis syndrome is associated with interchromosomal insertions mediated by a human-specific palindrome near SOX3. *American Journal of Human Genetics*.

[6] Rashid R. M., White L. E. (2007). A hairy development in hypertrichosis: a brief review of Ambras syndrome. *Dermatology Online Journal*.

[7] Eppig J. T., Richardson J. E., Kadin J. A., Ringwald M., Blake J. A., Bult C. J. (2015). Mouse Genome Informatics (MGI): reflecting on 25 years. *Mammalian Genome*.

[8] Hogan J., O'Connor C. T., Azizl A. (2012). Application of the Gene Expression Omnibus (GEO) to generate and validate consensus transcriptomic profiles that accurately differentiate nodal status in colorectal cancer. *Irish Journal of Medical Science*.

[9] Yi R., Poy M. N., Stoffel M., Fuchs E. (2008). A skin microRNA promotes differentiation by repressing ‘stemness’. *Nature*.

[10] Bello S., Smith C. L., Dene H. (2014). Integrating large-scale phenotyping data into the mouse genome informatics database. *Transgenic Research*.

[11] Carlson M., Falcon S., Pages H., Li N. org.Mm.eg.db: Genome wide annotation for Mouse.

[12] Carlson M., Falcon S., Pages H., Li N. (2013). *org. Hs. eg. db: Genome Wide Annotation for Human*.

[13] Barrett T., Edgar R. (2006). Gene expression omnibus: microarray data storage, submission, retrieval, and analysis. *Methods in Enzymology*.

[14] Van Peer G., Lefever S., Anckaert J. (2014). miRBase Tracker: keeping track of microRNA annotation changes. *Database*.

[15] Durinck S., Moreau Y., Kasprzyk A. (2005). BioMart and Bioconductor: a powerful link between biological databases and microarray data analysis. *Bioinformatics*.

[16] Osterhoff M. A., Frahnow T., Seltmann A. C. (2014). Identification of gene-networks associated with specific lipid metabolites by Weighted Gene Co-Expression Network Analysis (WGCNA). *Experimental and Clinical Endocrinology & Diabetes*.

[17] Langfelder P., Horvath S. (2008). WGCNA: an R package for weighted correlation network analysis. *BMC Bioinformatics*.

[18] Shannon P., Markiel A., Ozier O. (2003). Cytoscape: a software Environment for integrated models of biomolecular interaction networks. *Genome Research*.

[19] Bindea G., Mlecnik B., Hackl H. (2009). ClueGO: a cytoscape plug-in to decipher functionally grouped gene ontology and pathway annotation networks. *Bioinformatics*.

[20] Betel D., Wilson M., Gabow A., Marks D. S., Sander C. (2008). The microRNA.org resource: targets and expression. *Nucleic Acids Research*.

[21] Chou C.-H., Chang N.-W., Shrestha S. (2016). miRTarBase 2016: updates to the experimentally validated miRNA-target interactions database. *Nucleic Acids Research*.

[22] Wong N., Wang X. (2015). miRDB: an online resource for microRNA target prediction and functional annotations. *Nucleic Acids Research*.

[23] Gomez-Cabrero D., Abugessaisa I., Maier D. (2014). Data integration in the era of omics: current and future challenges. *BMC Systems Biology*.

[24] Vogel C., Chothia C. (2006). Protein family expansions and biological complexity. *PLoS Computational Biology*.

[25] Xia K., Fu Z., Hou L., Han J.-D. J. (2008). Impacts of protein-protein interaction domains on organism and network complexity. *Genome Research*.

[26] Vinogradov A. E. (2012). Human more complex than mouse at cellular level. *PLoS ONE*.

[27] Wu J., Husile, Sun H. (2013). Adaptive evolution of Hoxc13 genes in the origin and diversification of the vertebrate integument. *Journal of Experimental Zoology Part B: Molecular and Developmental Evolution*.

[28] Wu J. H., Xiang H., Qi Y. X. (2014). Adaptive evolution of the STRA6 genes in mammalian. *PLoS ONE*.

[29] Erwin D. H., Davidson E. H. (2009). The evolution of hierarchical gene regulatory networks. *Nature Reviews Genetics*.

[30] Mildner M., Jin J. A., Eckhart L. (2010). Knockdown of filaggrin impairs diffusion barrier function and increases UV sensitivity in a human skin model. *Journal of Investigative Dermatology*.

[31] Slominski A., Tobin D. J., Zmijewski M. A., Wortsman J., Paus R. (2008). Melatonin in the skin: synthesis, metabolism and functions. *Trends in Endocrinology and Metabolism*.

[32] Fuchs E. (2007). Scratching the surface of skin development. *Nature*.

[33] Gu L.-H., Coulombe P. A. (2007). Keratin function in skin epithelia: a broadening palette with surprising shades. *Current Opinion in Cell Biology*.

[34] Romanovsky A. A. (2014). Skin temperature: its role in thermoregulation. *Acta Physiologica*.

[35] Pasparakis M., Haase I., Nestle F. O. (2014). Mechanisms regulating skin immunity and inflammation. *Nature Reviews Immunology*.

[36] Folk G. E., Semken A. (1991). The evolution of sweat glands. *International Journal of Biometeorology*.

[37] Taylor D. K., Bubier J. A., Silva K. A., Sundberg J. P. (2012). Development, structure, and keratin expression in C57BL/6J mouse eccrine glands. *Veterinary Pathology*.

[38] Yi R., Fuchs E. (2010). MicroRNA-mediated control in the skin. *Cell Death & Differentiation*.

[39] Zou Q., Mao Y., Hu L., Wu Y., Ji Z. (2014). miRClassify: an advanced web server for miRNA family classification and annotation. *Computers in Biology and Medicine*.

[40] Gentner B., Schira G., Giustacchini A. (2009). Stable knockdown of microRNA *in vivo* by lentiviral vectors. *Nature Methods*.

[41] Brown B. D., Naldini L. (2009). Exploiting and antagonizing microRNA regulation for therapeutic and experimental applications. *Nature Reviews Genetics*.

[42] Liang T., Guo L., Liu C. (2012). Genome-wide analysis of mir-548 gene family reveals evolutionary and functional implications. *Journal of Biomedicine and Biotechnology*.

[43] Li Y., Xie J., Xu X. (2013). MicroRNA-548 down-regulates host antiviral response via direct targeting of IFN-*λ*1. *Protein and Cell*.

[44] Zhou R., Yang L., Liu C. (2013). Hsa-Mir-548l inhibits the invasion and metastasis of non-small cell lung cancer by targeting Erk1 And Pkn2. *American Journal of Respiratory and Critical Care Medicine*.

[45] Gu W., An J., Ye P., Zhao K.-N., Antonsson A. (2011). Prediction of conserved microRNAs from skin and mucosal human papillomaviruses. *Archives of Virology*.

[46] Dickson M. C., Ludbrook V. J., Perry H. C., Wilson P. A., Garthside S. J., Binks M. H. (2015). A model of skin inflammation in humans leads to a rapid and reproducible increase in the interferon response signature: a potential translational model for drug development. *Inflammation Research*.

[47] Zhang W., Zhang L. C., Zhou Y. (2015). Synergistic effects of BMP9 and miR-548d-5p on promoting osteogenic differentiation of mesenchymal stem cells. *BioMed Research International*.

[48] Jian Q., An Q., Zhu D. N. (2014). MicroRNA 340 is involved in UVB-induced dendrite formation through the regulation of RhoA expression in melanocytes. *Molecular and Cellular Biology*.

[49] Wang Q., Chow J., Hong J. (2011). Recent acquisition of imprinting at the rodent Sfmbt2 locus correlates with insertion of a large block of miRNAs. *BMC Genomics*.

[50] Mardaryev A. N., Ahmed M. I., Vlahov N. V. (2010). Micro-RNA-31 controls hair cycle-associated changes in gene expression programs of the skin and hair follicle. *The FASEB Journal*.

[51] Dahmke I. N., Backes C., Rudzitis-Auth J. (2013). Curcumin intake affects miRNA signature in murine melanoma with mmu-miR-205-5p most significantly altered. *PLoS ONE*.

[52] Druz A., Betenbaugh M., Shiloach J. (2012). Glucose depletion activates mmu-miR-466h-5p expression through oxidative stress and inhibition of histone deacetylation. *Nucleic Acids Research*.

[53] Segre J. A. (2006). Epidermal barrier formation and recovery in skin disorders. *The Journal of Clinical Investigation*.

[54] Li Y., Fan X., He X. (2012). MicroRNA-466l inhibits antiviral innate immune response by targeting interferon-alpha. *Cellular and Molecular Immunology*.

